# Breaking the silence – systematic review of the socio-cultural underpinnings of men’s sexual and reproductive health in Middle East and North Africa (MENA): A handful of taboos?

**DOI:** 10.1080/20905998.2024.2387511

**Published:** 2024-08-13

**Authors:** Walid El Ansari, Kareem El-Ansari, Mohamed Arafa

**Affiliations:** a Department of Surgery, Hamad Medical Corporation, Doha, Qatar; b College of Medicine, Qatar University, Doha, Qatar; c Department of Clinical Population Health, Weill Cornell Medicine – Qatar, Doha, Qatar; d Faculty of Medicine, St. George’s University, Saint George’s, Grenada; e Urology Department, Hamad Medical Corporation, Doha, Qatar; f Andrology Department, Cairo University, Cairo, Egypt; g Department of Urology, Weill Cornell Medicine-Qatar, Doha, Qatar

**Keywords:** Sexual, reproductive health, culture, religion, MENA

## Abstract

**Background:**

Sociocultural aspects can impact sexual and reproductive health (SRH). Despite this, no study appraised the socio-cultural underpinnings impacting men’s SRH in MENA (Middle East and North Africa). The current systematic review undertook this task.

**Methods:**

We searched PubMed and Scopus electronic databases for original articles on socio-cultural aspects of men’s SRH published from MENA. Data were extracted from the selected articles and mapped out employing McLeroy’s socioecological model. Analyses and data synthesis identified the factors impacting men’s experiences of and access to SRH.

**Results:**

A total of 53 articles were included. Five related socio-cultural underpinnings intertwined with taboos were observed that affect three main SRH topics, HIV, reproduction and sexuality across three broad population groups: HCP/health services, school/university students, and the general public/patients. These underpinnings included 1) Challenges to gender equality; 2) Religious prohibitions and misinterpretations; 3) Sexual rights and taboos; 4) Masculinity and manhood ideals; and 5) Large families and consanguinity. In terms of research, a paradox exists, as we found virtually no research on four socio-cultural underpinnings of men’s SRH in MENA pertaining to: a) other STI, despite being common; b) other features of reproduction, despite that religio-cultural factors play a critical role; c) sexuality, despite the high prevalence of sexual disorders, and, d) gender-based violence, despite the widespread partner violence.

**Conclusions:**

Socio-cultural underpinnings are deeply rooted across MENA population groups including HCPs, students, general public, and patients with negative impact on the perceptions and dealings pertaining to men’s SRH issues including HIV, reproduction and sexuality. The findings call for concerted widespread efforts to enhance the socio-cultural acceptance of these population groups while highlighting any misinterpretations of religious rules pertaining to men’s SRH. Moreover, breaking the silence on such issues necessitates more enthusiasm across MENA health systems, with future research examining the effects of such efforts on the socio-cultural aspects of men’s SRH in MENA.

## Introduction

The Arab region is home to more than 400 million people [1], with males accounting for about 52% of the population [2]. Young people between 15 and 29 years of age constitute nearly 24% of the population, compared with 19% of the population across the Organization for Economic Cooperation and Development countries [3]. The Middle East and North Africa (MENA) include a group of nations and populations that share similar language, culture, and traditions [4]; and culture is a principal concept when it comes to understanding sexual behaviors and better sexual and reproductive health (SRH) [5]. Sexual health is a state of physical, mental and social well-being in relation to sexuality [6] and good SRH is complete physical, psychological and social integrity in all matters related to the reproductive system [1].

MENA experiences a range of men’s SRH-related challenges. For instance, the prevalence of erectile dysfunction (ED) in Arab countries is suggested to be high, although direct evidence is lacking [7]. In addition, although the HIV/AIDS epidemic has declined in most world regions, its incidence in MENA is still rising [8,9], with a male/female ratio of 4:1 [10]. Equally, other STI are prevalent, where for example, in the Kingdom of Saudi Arabia (KSA), between 2005 and 2012 there were 68,886 new cases of STIs, of which non-gonococcal urethritis was the highest [11].

Despite such a state of men’s SRH in MENA, sexuality remains encompassed with feelings of utmost sensitivity and privacy, as religious norms emphasize the secrecy of one’s sexual matters [12,13]. Indeed, conversations in MENA about sexuality are largely taboo or, at minimum, impolite [14]. Men and women alike may not be forthcoming in revealing their personal sexual behavior, and it is certainly more so in MENA [5], as evidence suggests considerable socio-cultural sensitivities about SRH and stigma/misconceptions attached to SRH conditions and the individuals living with it. Similarly, the blanket of silence and denial encompassing STIs in MENA society means that young people might have never heard of or discussed them, nor even seen or known a person suffering from a STI [15]. As a result, culturally appropriate and accurate data pertaining to men’s SRH in MENA is lacking [16,17].

Engaging men in SRH is important for many reasons: the prevention of sexually transmissible infections; promoting healthy relationships and behaviours, including the treatment of male sexual dysfunction; optimising fertility; and improving the chances of a healthy pregnancy and child [18]. Engaging men in SRH is also an opportunity to promote general wellbeing and to engage in conversations about overall health and the prevention of future illness [18].

Collectively, these notions acted as the driver of the present systematic review that aimed to explore the current socioculture-specific considerations that impact on men regarding SRH in MENA. The specific objectives of the review were to a) identify the population groups affected by such socioculture barriers; b) explore the main SRH domains that are most impacted by socioculture traditions and restrictions; c) characterize and summarize the actual socio-cultural taboos that underpin and reinforce the current notions and silence around men’s SRH in MENA; and d) appraise the knowledge gaps in socio-cultural research addressing men’s SRH in MENA. In addition, we explored good practices that could be effective for overcoming socio-cultural barriers to better men’s SRH in MENA. Incorporating SRH culture-specific considerations across various male population groups across MENA may improve preventive and intervention efforts to optimize outcomes at clinical practice and population levels.

## Methods

### Search strategy

A detailed systematic literature review was undertaken in accordance with the Preferred Reporting Items for Systemic Reviews and Meta Analyses (PRISMA) guidelines [19]. The search was performed using the electronic databases of PubMed and Scopus from inception until 1 June 2023. Searches were structured using Boolean operators combined keywords and database subject terms for SRH, specific for the MENA region and countries (Supplementary Box 1). We took MENA to include a group of countries that share similar language, culture, and traditions [4]. Similar to others [20], the searches used truncation to find variant word endings, such as Arab* for Arab, Arabs, Arabian, or Arabians. When necessary and possible, the searches included subject explosions, a database feature that simultaneously searches for a broad concept such as MENA region as well as the individual countries’ names.

### Inclusion/Exclusion criteria

Studies considered for inclusion had to address socio-cultural aspect/s of men’s SRH in a MENA country. The results were limited to original articles published in English language. Given the focus on socioculture-specific considerations that impact on men regarding SRHC in MENA, studies were excluded if they did not address social/cultural issues that could influence cultural care, e.g. technology; religion/philosophy; kinship; cultural values, beliefs, and lifeways; politics; economy; and education, in addition to psychosocial factors [21]. Review articles, case reports or editorials/correspondence were also excluded.

### Selection of studies for inclusion

Using a checklist, two authors (WEA, MA) independently screened potentially eligible articles that met the inclusion/exclusion criteria. The preselected studies were agreed upon, and discrepancies were resolved by consensus. Accepted articles were carefully read, and their reference lists were examined to identify possibly relevant articles not located in the initial search.

### Definitions

Table 1 depicts the definitions of selected terms used in the present systematic review.

**Table 1.** Definitions of selected terms.

Term	Meaning
SRH	Good sexual and reproductive health is a state of complete physical, mental and social well-being in all matters relating to the reproductive system. It implies that people are able to have a satisfying and safe sex life, the capability to reproduce and the freedom to decide if, when, and how often to do so [22]
Sexuality	Central aspect of being human throughout life encompasses sex, gender identities and roles, sexual orientation, eroticism, pleasure, intimacy and reproduction. Sexuality is experienced and expressed in thoughts, fantasies, desires, beliefs, attitudes, values, behaviours, practices, roles and relationships, and influenced by the interaction of biological, psychological, social, economic, political, cultural, legal, historical, religious and spiritual factors [1,6]
Sexuality Education	Knowledge of a broad range of issues relating to the physical, biological, emotional and social aspects of sexuality [1,6]
Sexual Orientation	Refers to an enduring pattern of emotional, romantic, and/or sexual attractions to men, women, or both sexes [1,6]
MENA	Includes Algeria, Bahrain, Egypt, Iraq, Jordan, Saudi Arabia, Kuwait, Lebanon, Libya, Morocco, Oman, Palestine, Qatar, Sudan, Syria, Tunisia, United Arab Emirates (UAE), Yemen, Somalia, Djibouti, Western Sahara, Mauritania [4].
Culture	A pattern of ideas, customs and behaviours shared by a particular people or society, it is constantly evolving [23]. It refers to the sum total of acquired values, beliefs, practices, laws, customs, traditions, artifacts and knowledge possessed and expressed by a designated group, or ‘all human nongenetic, or metabiological, phenomena’ [24]
Religion	A cultural system of behaviours and practices, world views, ethics, and social organization that relate humanity to an order of existence. About 84% of the world’s population is affiliated with one of the five largest religions namely Christianity, Islam, Hinduism, Buddhism or folk religion [25–27]
Religiosity	Refers to the behaviour and attitude associated with a level of commitment to the beliefs and practices of a faith tradition [28]
Stigma	Social construct that singles out a person by virtue of a physical or social trait, resulting in negative social reactions such as discrimination and avoidance [29]

MENA: Middle East and North Africa; SRH: Sexual & reproductive health.

### Framework

**Figure 1.**
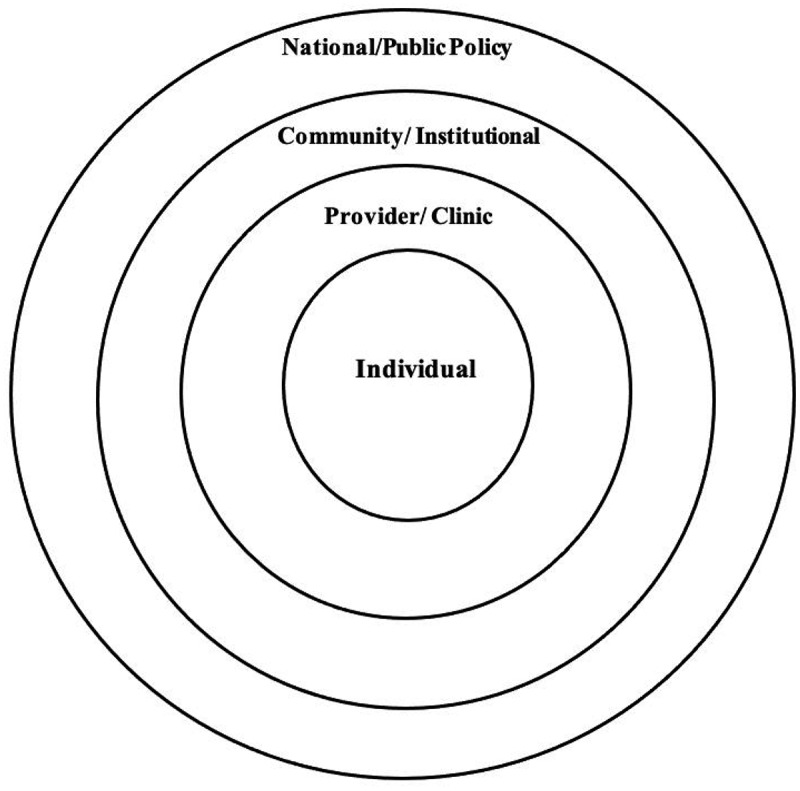
Socioecological model showing the cultural-specific factors influencing men’s sexual and reproductive health [30].

The current review was guided by the socioecological model framework that focuses on both the individual and social environmental factors, including the interpersonal, organizational, community, and public policy, factors that support and maintain unhealthy behaviors [30]. The model assumes that appropriate changes in the social environment will produce changes in individuals and that the support of individuals in the population is essential for implementing environmental changes (Figure 1).

## Results

### Search results

**Figure 2.**
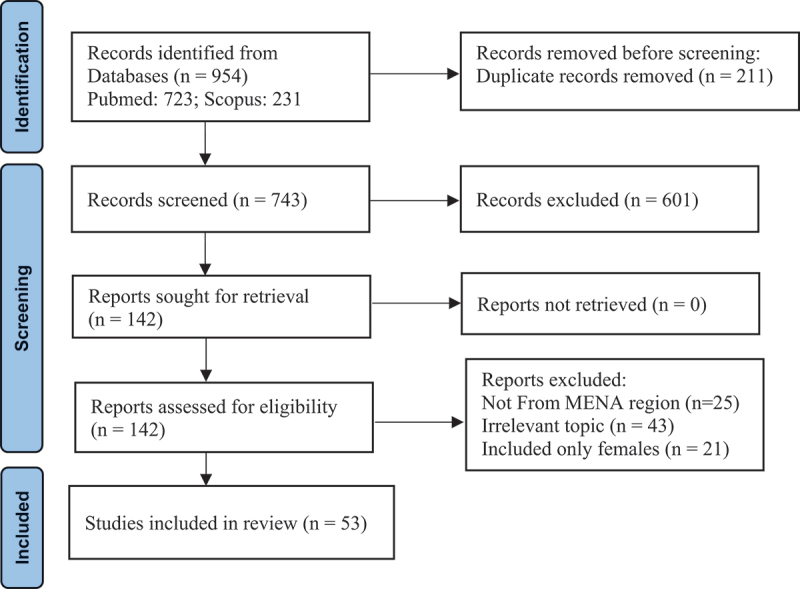
PRISMA flow chart on search results of socio-cultural aspects of men’s SRH in MENA countries [19].

Figure 2 shows the PRISMA flow chart of search results of socio-cultural aspects of men’s SRH in MENA countries. A total of 53 articles were finally included in the present review.

### Characteristics of the sample

The 53 studies (Supplementary file 1) that the review identified included three population groups: 11 articles (20.75%) involved school/university students, 23 articles (43.4%) focused on healthcare professionals (HCP), and 19 articles (35.85%) were related to patients/general public. With regard to the topic of men’s SRH discussed, the majority of studies (29 articles, 54.72%) focused on HIV. Fertility and sexuality issues were independently addressed in 13 (24.53%) and 9 (16.98%) articles, respectively, while 2 articles (3.77%) targeted both fertility and sexuality issues combined. The highest number of socio-cultural research originated from Jordan (12 studies, 22.64%), KSA (11 studies, 20.75%) and Egypt (8 studies, 15.09%), followed by Yemen (*n* = 5), Lebanon (*n* = 4), Oman, Kuwait and United Arab Emirates (3 articles each). The least contributions were from Sudan, Somalia, and Iraq (1 article each), while one study was a joint collaboration between Yemen and Sudan. The current review identified no studies from Algeria, Bahrain, Djibouti, Libya, Morocco, Qatar, Syria, Tunisia, Palestine, and Western Sahara.

### Socio-cultural findings

The data extracted from the 53 articles are presented in Table 2. The data highlight the range of culture-specific considerations surrounding a range of issues pertaining to men’s SRH in MENA. We display the emerging data in accordance with an ecologic perspective, focusing on people’s interaction with their physical and sociocultural environment [30,80] and in tandem with the World Health Organization’s (WHO) operational approach to sexual health and its linkages to reproductive health [81]. The section below depicts the findings by the population examined (HCP/health services, students, general public/patients) across SRH topics.

**Table 2.** Socio-cultural findings of men’s sexual and reproductive health in MENA by population group, issue, and country.

Parameter	Population group		
	*HCP/Health Services*	School/University Students	General Public/Patients
HIV	**▶Blame, Shame, Stigma** • *By HCPs*: in Egypt, Sudan, Yemen and KSA, religiously rooted beliefs that PLWHA should be ashamed of themselves, should be blamed for their infection, with suggestions of isolation of HIV cases in specialized hospitals/facilities [31–34] • *Against HCPs*: in Egypt, stigma extended to physicians providing care for PLWHA, caused by fear of infection, to the extent of community unwillingness to use those physicians’ services. On the other hand, stigma toward physicians who refused to provide care was linked to perceptions of unethical behavior [35]	**▶ Blame, Shame, Stigma** • *Yemen, KSA, UAE*: neither friendly nor tolerant toward PLWHA, high level of stigma, shame, discrimination, victimization and taboo to extent of even discussing the topic with others, believed that PLWHA needed to be punished and isolated, to the extent of suggestions of killing PLWHA [36–41]	**▶ Blame, Shame, Stigma** • *Jordan*: decreased adherence to HIV medications was related to stigmatization [42] • *Somalia*: PLWHA faced such stigma in their homes and at work, forcing them to seek support from NGOs or close family. Stigma affected their disclosure to the wider community due to uncertainty of the repercussions, leading to a lonely life [43] • *Oman*: a common sense of fatalism existed regarding HIV acquisition [5] • *Yemen*: Stigma and intolerance towards PLWHA [44,45]
	**▶Culturally-based Non-Acceptance Attitude Towards PLWHA** • Evident across MENA countries e.g. KSA [31,46,47], Kuwait [48], Jordan [49], Egypt [32], Oman [50]		
	**▶ Disapproving Attitudes to PLWHA by Different HCPs** • *Physicians*: in Kuwait, majority preferred not to treat PLWHA or even come in contact with HIV sero-positive cases [48] **•** *Dentists*: in KSA, may refuse to treat PLWHA [46] • *Nurses*: in Jordan and Egypt, refuse or prefer not to be involved in treatment of PLWHA [32,49]		**▶ Disapproving Attitudes** • *Lebanon*: HIV testing uptake was limited by concerns about the confidentiality and fear of repercussions on health and employment [51]
	**▶ Culturally-based restrictions Towards HIV Prevention** • *Sudan, Yemen*: unfavourable religious context and social and cultural environment many restrictions on HIV prevention [34]		
	**▶Cultural Acceptance** • *Kuwait*: MoH dental assistants displayed more positive attitude towards PLWHA [52] • *KSA*: adequate moral beliefs reflecting professional ethics were associated with lower odds of refusal to treat PLWHA [46] • *Egypt*: context-specific and culturally appropriate HIV stigma-reduction interventions can reduce value and cultural-based stigma among physicians, nurses and health authorities [33,35,53]	**▶ Cultural Acceptance** • *Interactions*: in Oman, students or colleagues with the HIV infection attending the same classroom and working place were accepted by majority of medical and non-medical students [54] • *Care for PLWHA*: in Yemen, more recently, moderate acceptance of PLWHA, as most students were willing to care for PLWHA [38] • *Education interventions*: in Yemen, school-based peer education intervention decreased stigma and discrimination levels [55]	• *Kuwait*: Most subjects thought that religion was important in dealing with daily life problems e.g. HIV infection [56]
Reproduction	**▶Fertility Preservation** • *Gender*: in Lebanon, significantly more male than female patients informed of their fertility preservation options before cancer treatment [57] • *Religion*: in Egypt and KSA, it was not a critical factor affecting oncologists’ low perception of the importance of cryopreservation [58,59]		
		**▶Premarital Screening/Consanguinity** • *KSA*: Premarital screening generally accepted by university students to prevent disease transmission to offsprings and ensure partner’s wellbeing; fair number willing to change their decision to marry in case of receiving incompatible results; majority demanded implementation of law prohibiting incompatible marriages [60] • *Oman*: adolescent boys favored marriage at younger age, polygamy, having many children, consanguineous marriage [61]	**▶Premarital Screening/Consanguinity** • *Egypt*: public had favorable attitudes, probably related to social changes in village life leading to delay in beginning a family [62]
	**▶Reproductive Services for Youth** • *Nurses*: viewed reproductive health questions by youth as inappropriate, treating them like children with grim faces [63] • *Physicians*: did not communicate in good way with youth, rarely smiled and did not take young person’s problems seriously [63]		
		**▶Contraception** • *KSA*: cultural and religious unacceptability negatively influenced knowledge of EC [64] • *Oman*: most adolescent boys agreed with use of modern contraceptive methods in future [61]	**▶Male Contraception/Gender bias** • *UAE*: religious/cultural barriers, personal beliefs were main reasons for male contraception being not accepted by general public [65] • *Sudan*: most men interested in learning about female > male sterilization [66] • *Iraq*: condoms rarely used for family planning, preference for female contraception [67] • *Jordan*: Almost half the men in Jordan did not believe in family planning, viewing that family size was up to God [68,69]. Likewise, pharmacists expressed that cultural and social norms were barriers to male contraception use [70] • *KSA*: majority of men believed that contraception methods are not prohibited in Islam [71]
Sexuality	**▶ Sex Education** • *Nurses*: in Egypt were influenced by their beliefs about sex education and attitudes regarding their own family/children and sexual development [72,73] • *Nurse’s gender*: in Jordan, this influenced sexual counseling, where males exhibited higher confidence and practice than females [74] • *Physician’s gender* - *KSA*: social and cultural factors accounted for the significant gap in knowledge and interest towards ED among female physicians in KSA. Physicians most often serviced same sex clients [75]. - *Jordan*: more male HCP seeing male clients as compared to female HCP for issues related to puberty, biological concerns, STIs, condoms, information about sexual activity, romantic relationships between partners [76]	**▶ Sex Education** • *Rights*: due to MENA conservative culture, adolescents are sometimes not considered to have the right to sexuality information [55] • *Female genital cutting*: in Oman, adolescent boys considered female genital cutting a necessity [61] • *Liberal attitude*: Lebanon, despite social-culture considerations, university students had liberal attitude towards sex and male sex [77]	**▶Sex Education/Counselling** • *Shame*: in KSA, ED is sensitive issue, about 28% of men with ED consulted their physician or used herbal and over the counter drugs [12] • *Gender/Shame*: in Jordan, most Arab-Muslim cardiac patients preferred that the cardiologists provide them with sexual information, while two-thirds considered nurses’ gender to be a barrier preventing them from inquiring about their sexual life [14] **▶Erectile Dysfunction** • *KSA*: shame, socio-religious concerns, and embarrassment were reasons for overlooking ED as a health disorder, therefore do not seek medical advice [12,75]
	**▶ LGBT** • *HCP*: in Lebanon, generally showed positive attitudes and behaviors toward LGBT patients, more favorable now when compared to previous investigations [78] • *Mental health providers*: in Lebanon, more receptive compared to non-mental health providers and more willing to address transgender people by their gender pronouns [78]		▶ **LGBT** • *Shame*: in Egypt, guilt feelings about trial to change sexual orientation were very high, associated with high religiosity, most participants did not disclose their sexual orientation except to their partners and will not disclose it to healthcare providers even if needed [79]

KSA: kingdom of Saudi Arabia; UAE: United Arab Emirates; PLWHA: people living with HIV/AIDS; HCP: healthcare professionals; LGBT: lesbian, gay, bisexual, and transgender; ED: erectile dysfunction; EC: emergency contraception; HIV: Human Immunodeficiency Virus; AIDS: Acquired Immunodeficiency Syndrome; MoH: Ministry of Health; MENA: Middle East and North Africa.

#### HCP and health services

##### HIV

Table 2 depicts the effect of cultural factors on HCP in relation to people living with HIV/AIDS (PLWHA). Generally, across many MENA nations, HCP generally expressed stigmatization and avoidance towards PLWHA, probably due to their conservative cultural backgrounds and religious beliefs leading to moral judgement towards homosexuality and PLWHA. Such disapproving attitudes spanned the different categories of HCPs, including physicians, dentists and nurses, although expressing such stigma was less for physicians, expatriates and those with more SRH experience [31,32,46–50]. Nevertheless, exceptions also existed in some MENA countries with more favorable attitudes towards PLWHA based on professionalism and aided by stigma-reduction interventions [33,35,46,52,53]. Conversely, in Egypt, stigma *against* HCP who provide care for PLWHA was also reported [35].

##### Reproduction

Social norms in MENA influenced the HCP’s dealings with a number of male reproductive issues (Table 2). Culturally-based gender bias was observed in Lebanon pertaining to the provision of fertility preservation information to clients prior to cancer therapy, where HCP provided information to significantly more male clients than females [57]. Conversely, religion was not viewed as an important predictive factor underpinning the oncologists’ low perception of the importance of cryopreservation in Egypt and KSA [58,59]. Conservative cultural attitudes also led to reservations when HCP discussed reproductive issues with youth [63], and youth reproductive services were unfriendly to the youth in Jordan as nurses and physicians exhibited repulsive attitudes [63]. However, physicians and HCP with previous SRH training exhibited more youth-friendly mindsets than nurses [82].

##### Sexuality

Cultural and moral beliefs as well as personal and family experience of the HCP with their own sexual development shaped their approach to sexual counselling. For instance, some nurses believed that starting sex education too early could be a problem, and at times, this was linked to a sense that sex education represents a cultural change or even a threat that parents may see as adversely affecting their children [72,73]. Gender norms were found to drive sexual counseling in the MENA region, e.g. in Jordan and KSA, male HCP displayed higher confidence in practicing sexual counselling than females, and physicians most often serviced same sex clients when delivering SRH services [74–76]. Among some more culturally liberal MENA countries, e.g. Lebanon, HCP and particularly mental health providers generally showed positive attitudes and behaviors toward LGBT patients [78]. Such standpoints were more favorable when compared to the findings of earlier investigations in Lebanon [78].

#### Students (schools and universities)

##### HIV

Table 2 also illustrates the cultural and religious considerations associated with students’ attitudes towards PLWHA. Cultural values and beliefs that PLWHA were responsible for their own infection, and religious views that AIDS is a punishment from God led to negative feelings towards PLWHA among school and university students in Yemen, KSA and UAE [36–41]. Conversely, students in some MENA countries displayed higher levels of cultural acceptance, probably due to school-based peer education interventions that were implemented [38,54,55].

##### Reproduction

Cultural norms seemed to affect students’ ideas towards reproductive issues (Table 2). Societal and culturally inclined beliefs towards marriage including marriage at a younger age, polygamy, large family size, and consanguineous marriage were evident among adolescent boys in Oman, despite that the majority agreed with the use of modern contraceptive methods in the future [61]. Conversely, in KSA, where consanguineous marriages are very common, a knowledge-based cultural change was observed among university students, where premarital screening was generally being accepted to the extent of their willingness to legalize prohibition of incompatible marriages [60].

##### Sexuality

Due to MENA conservative culture, adolescents were sometimes not viewed to have the right to sexuality information [55]. Moreover, in Oman, students felt that female genital cutting was a necessity [61]. Conversely, despite social-culture considerations, in the more progressive countries, e.g. Lebanon, students had liberal attitude towards sex and male sex [77].

#### General Public/patients

Cultural values and religious beliefs profoundly influenced the attitudes of patients as well as the general public pertaining to a range of male SRH issues (Table 2) as outlines below.

##### HIV

In Jordan, shame and fear of stigmatization led to low patient’s adherence to HIV medications [42]; in Somalia, PLWHA faced stigma at home and at work [43]; in Oman, HIV acquisition had a very miserable overcast [5]; and in Lebanon, HIV testing uptake was limited by fears about confidentiality [51].

##### Reproduction

Similarly, cultural beliefs underpinned the low acceptability of the use of male contraception in MENA [65–67,70]. Sometimes, a paradox was observed between socially rooted beliefs surrounding SRH and the actual religious facts. On the one hand, socio-cultural barriers underpinned the low acceptance of male contraception, e.g. in UAE, Sudan and Iraq [65], with more interest in female sterilization and contraception [66,67]. There was also low contraceptive use by married men in Jordan, viewing that family size was up to God, despite the positive attitudes and good knowledge about family planning [68]. On the other hand, in KSA, the majority of men believed that contraception methods are not prohibited in Islam [71]; and social transformations in village life in Egypt led to changes in cultural behaviors with more inclination towards delaying the beginning of a family, which reflected positively on premarital counselling, as the general public displayed favorable attitudes towards such services [62].

##### Sexuality

Cultural constraints underlie the shame surrounding SRH issues in MENA. For instance, in KSA, erectile dysfunction was not viewed as a health disorder, rather a sensitive issue that led to the unwillingness of patients to discuss sexual disorders even with doctors [12,75]. Similarly, in Jordan, Arab cardiac patients considered nurses’ gender to be a barrier preventing them from inquiring about their sexual life [14]. Pertaining to LGBT, in Egypt, high religiosity, shame and guilt feelings about trial to change sexual orientation were very high [79].

## Discussion

‘The consequences of stepping outside traditional behaviour are so severe that behaviour may not change even if knowledge and attitudes do’. [83]

An individual’s life processes are strongly associated with social and cultural factors that exist in political and managing contexts, as well as the perspectives of health and disease [84]. All cultures have systems of health beliefs to explain what causes illness [85], and many health conditions are embroiled in pertinent cultural contexts. An example in mental health is schizophrenia, where cultural prejudgments of conduct are common in social practice and the health care that is provided [86]. Likewise in obesity, a deeper understanding requires considerations of the cultural context of food-related health behaviors [87]. In a similar manner, SRH is deeply rooted in culture.

Arab societies share relatively comparable cultures and religious conservatism [88]. Hence, discussing SRH issues is regarded as taboo among the general public, frequently characterized by no formal sexual education [89]. Traditionally, SRH has been examined from the perspectives of women and feminist scholarship and activism [90]. To our knowledge, this is the first review to undertake an in-depth examination of the interlacing socioculture underpinnings that enmesh SRH from the perspectives of MENA men and the impact of such considerations on men’s SRH care, along with proposing a range of relevant solutions for the way forward.

Our main findings are that men’s SRH in MENA face the combined influences of cultural values and constraints, religious sensitivities, community norms, embarrassment and shame, and secrecy and stigma. Collectively, these customs, norms, beliefs and values act as an underlying covert restraining ‘filter’ that influences SRH perceptions, attitudes and service utilization. Such mindsets were apparent across the population groups we examined, namely HCP, school and university students, and the general public/patients, and across many SRH issues. Our findings fall under five main categories influenced by the prevailing cultural norms in MENA. Below, we detail each.

### Socio-cultural factors

#### Challenges to gender equality

The current review found that gender bias favors men. This influenced the SRH practices of HCP, the preferences of patients, and the beliefs of the general public in terms of fertility preservation and also sexuality. For instance, oncologists were more inclined to inform male rather than female patients of their fertility preservation options before cancer treatment [57]. Equally, in Sudan, Iraq and UAE, most of the general public and patients were biased towards the practice of female rather than male contraception [65–67]. Likewise, female genital cutting was widely accepted by adolescent male students in Oman [61]. Such findings resonate with the sociological notion of patriarchal systems.

Indeed, most African societies and MENA nations operate a patriarchal system with female submission in social relations and marriage, challenging the notions of gender equality [91]. A recent MENA survey found that ‘traditional’ attitudes about gender equality still prevail, including the younger generation men [92]. Given that men in MENA have not traditionally shared equal responsibility for fertility regulation [65], it would be valuable for HCP across MENA to realize that sexual counselling and SRH issues are indeed concordant with Islamic teachings [74], contrary to popular community beliefs prevailing across many MENA societies.

#### Religious prohibitions and misinterpretations

Most MENA populations are of the Muslim faith, with about 4% Christians [93], mostly in Lebanon, Egypt, Syria and Jordan [94]. Out-of-marriage sexual relationships are prohibited according to religious and social codes in Islam and Christianity alike [83,95]. Within the same vein, globally, religious beliefs and affiliation are powerful predictors of attitudes about homosexuality [96].

Such religious prohibitions might explain our observations of why public discussions of STIs, HIV and AIDS, which are commonly linked to extra-marital relations and homosexuality, remain a taboo in MENA. This was associated with the widespread socio-cultural non-acceptance and stigmatization attitudes of HCP and students towards PLWHA [39,49]. This is despite the estimated half a million PLWHA across MENA [97]. These societal views towards PLWHA were also reported across other cultures [98]. Globally, such stigma toward PLWHA among HCP represents a fundamental barrier to effective prevention and health care [99].

In the current review, religious misinterpretations were observed among school and university students in KSA and Yemen, who felt that AIDS is a punishment from God [36,39]. Such a viewpoint concurs with other religions, where different Christian churches reported that ‘people still interpret HIV/AIDS as a punishment from God, attaching PLWHA with immoral behaviors’ [100]. Similarly, among South African multi-ethnic communities, religiously based stigma towards PLWHA arose from people’s personal beliefs and justification that PLWHA did not adhere to religious teachings and injunctions [101].

HIV-related religious stigma can inflict hardship and sufferings upon PLWHA and interferes with their counseling/testing-seeking decisions, observed in MENA as well as other cultures [42,98]. Evidence on barriers to HIV/AIDS and STI testing among Muslims is limited [102], making it difficult to draw conclusions; but nevertheless provides insight into hindrances specific to conservative Muslim cultures.

#### Sexual rights and taboos

Sexuality is a complex phenomenon [103], and more so in MENA. For conservative Muslim communities, it is challenging to break the silence around matters of sexual behaviors, particularly those that diverge from religious norms [104]. Cultural, religious, and social factors may interfere with a willingness to discuss sexual issues [83,105] rendering them taboos. Sexual health is not taught in any formal setting in most Islamic countries. For instance, teachers at schools are hesitant to debate sensitive issues with students, constrained by socio-cultural inhibitions [106,107]. Likewise, many Muslim leaders, parents and young people are concerned about current methods of sex education and the values behind them, with the opinion that it is in serious conflict with Islamic teaching [108].

The current review observed that taboos pertaining to SRH may affect the sexuality rights of the population, evident in the case of sex education, where young people were not considered to have the right to sexuality information [55]. This was reflected by the negative attitudes of nurses and physicians, and such behaviors rendered youth reproductive services unfriendly to the youth [63]. Others have proposed that such culturally based sexuality sensitivity that encases young people might be due to the fact that many MENA countries fail to prioritise SRH, despite the many supporting initiatives that voice the need to educate young Muslims on these issues [109].

Sexual taboos were not limited to males, as others similarly found that explicit discussions between Muslim mothers and daughters about marriage and sex were surrounded with embarrassment [110]. Cultural taboos also underpinned the perspectives of adolescents regarding sexual rights of the opposite sex, as we observed that adolescent boys in Oman genuinely considered female genital cutting a necessity [61].

We also observed that sexual taboos across MENA extended to HCPs, underlining their counselling and practice, where female HCPs exhibited lower confidence and interest in dealing with men’s sexual dysfunctions, preferring same sex clients [74–76]. Such findings resonate with Turkey, which has near similar socio-cultural perspectives, where nurses did not initiate dialogue about sexual concerns with patients [111]. This is in contrast to nurses from European countries who were knowledgeable and comfortable discussing patients’ sexual concerns [112]. Such differences confirm that culture certainly matters when it comes to sexuality [113].

#### Concepts of masculinity and manhood ideals

If discussing sexual concerns in certain non-MENA societies is a taboo [114], then, for Arab societies, the fear of being impotent may predominate over all other fears [115]. The current review is in agreement, where MENA socio-cultural masculinity and manhood ideals led to embarrassments in discussions around sexual conditions. In KSA, erectile dysfunction was not viewed as a health disorder, but rather, a sensitive and shameful issue [12,75], leading to a reluctance of patients to discuss such conditions with their physicians and relying instead on herbal remedies [12]. This is unfortunate, as ED detection/treatment could be critical, given that it is a sentinel marker of overall men’s health and particularly cardiovascular diseases [116].

Masculinity ideals across MENA added another layer of embarrassment pertaining to men’s discussions about SRH with the opposite sex. The present review noted that across the Arab nations, male patients viewed HCP’s gender as a barrier preventing them from inquiring about their own sexual life [14]. Within the same context but with regard to females, Arab women may avoid talking about sex, especially with men, because of fears of losing their chastity or being described as ‘“fallen women”’ [83]. Such sentiments highlight the importance of HCPs preserving modesty while conversing sensitive issues, by using effective communication skills and appropriate language, rendering them more assertive and positive when practicing sexual counselling and honorably engaging with men concerning SRH issues [74].

Perhaps with SRH as a culturally sensitive topic, it could prove advantageous to note that the holy Quran and Hadith (Prophet Mohammad’s sayings) have discoursed remarkably private matters, e.g. intercourse and foreplay [13]. Embarrassment did not deter old Muslims, including women, to seek guidance from Prophet Mohammad about their sexual issues [13]. Thus, religious leaders should be involved as change agents, and their involvement can bring additional attitude change in the social norms and practice.

#### Large families and consanguinity

The interweaving of Islam and cultural background plays a powerful role in the lives of MENA populations, where large families are encouraged, to the extent that some scholars might prohibit contraception use [65]. The current review found that in some countries, contraception and family planning were not well received, as family size was viewed as God’s will [68]. For instance, adolescent boys still favored marriage at a younger age and polygamy, as having many children was valued in preparation for large families [61]. Condom use has been socially related to unfaithfulness, and some religious Muslim leaders could carry misconceptions about its use, equating such use with promoting sin [117]. This is in agreement with our findings that, in Iraq, condoms were rarely used for family planning [67].

### Breaking the silence – men’s SRH-related cultural changes across MENA

In contrast to the above, the current review also uncovered many favorable SRH-related cultural changes across some MENA countries, namely Kuwait, KSA, Egypt, Oman, Yemen and Lebanon.

In terms of HIV, we observed increasing awareness and acceptance towards PLWHA. These cultural changes were observed specifically among two population groups. The first were students, probably due to stigma reduction interventions and school-based peer education anti-discrimination programs [38,54,55]. The second was among HCP, probably rooted in their professional ethics, practice moralities, and heightened sense of duty and aided by stigma reduction interventions [33,35,46,52].

As for reproduction issues, we observed cultural changes pertaining to two SRH aspects. The first was the increased acceptance of family planning, probably influenced by modernization and changes in village life leading to delays in starting a family [62], coupled with a rising awareness that contraception methods are not prohibited in Islam [71]. The second was related to premarital screening, which was generally increasingly accepted by university students, as their knowledge and awareness towards the potential negative effects of consanguinity increased [60] and their willingness to safeguard the health of their future families.

Regarding sexuality issues, we observed cultural changes in the more liberal communities pertaining to two SRH aspects. The first was the expression of positive attitudes and behaviors toward LGBT, observed across HCP and students [78]. The second was the more liberal sexual behavior among university students, although others noted that such liberal attitudes increased students’ sexual risk-taking behavior, necessitating more awareness programs [77].

### Research on socio-cultural aspects of men’s SRH across MENA

The current review identified shortcomings in relation research on the socio-cultural aspects of men’s SRH across MENA. In terms of volume, we identified only 53 studies despite the critical importance of the topic for the region, although our search included several electronic databases and was not time-bound.

In relation to SRH topics, we found that the majority of the research revolved mainly around HIV, with some publications on family planning, premarital screening, erectile dysfunction and sex education. The current review found virtually no research on men’s SRH in MENA examining, for instance, the socio-cultural aspects pertaining to: a) other STI, despite that these, collectively, are more common than HIV [118]; b) other aspects of reproduction, e.g. assisted reproductive techniques (ART), offspring sex selection, male fertility/infertility, despite that the delayed establishment of ART centers for infertility treatment in MENA until the mid 1980s was mostly due to religio-cultural factors, as religious groups did not accept a third party’s involvement in procreation [119]; c) sexuality, e.g. sexual disorders other than ED, despite the high prevalence of, e.g. premature ejaculation in MENA [120], psycho-sexual development, despite the increasing popularity of such gender issue in the current climate; and d) gender-based violence, despite the high prevalence of intimate partner violence across Arab countries [17,121].

With regard to SRH research tools, no culturally appropriate standardized questionnaire has been developed to measure, e.g. HIV prevention knowledge in the MENA populations [122]. Until very recently, due to socio-cultural reasons, it has been difficult for researchers to incorporate attitudes and practices towards STIs in their investigations, sufficing to inquire only about knowledge of STDs among young adults [123]. Indeed, the majority of sex research among young people has been carried out in Western countries, and the findings are not easily transferable to Arab societies precisely due to cultural differences [83].

In terms of the geographic spread of research pertaining to the socio-cultural aspects of men’s SRH, the current review identified no studies from Algeria, Bahrain, Djibouti, Libya, Morocco, Qatar, Syria, Tunisia, Palestine, and Western Sahara. Publications in parallel fields reveal quite a similar picture. For instance, a bibliometric analysis of rheumatology research across the Arab World reported no contributions from Somalia, Mauritania, Djibouti, Palestine and Libya [124]. Others have similarly alluded to similar findings [125].

Hence, our findings concur with calls for future better-quality research on the socio-cultural underpinnings of health behaviors and motivations affecting men’s SRH [64]. Men’s engagement with SRH in MENA is essential now more than ever, given that young people are the fastest growing segment among these populations, and as each generation enters childbearing years in greater numbers, generating a larger number of births, a phenomenon referred to as ‘population momentum’ [126,127]. Others similarly noted that insufficient research due to cultural norms was widespread [128]. In addition, research would also benefit from more exploration of the identified variations within MENA countries to provide a deeper understanding of the reasons behind such variations.

### A way forward

**Figure 3.**
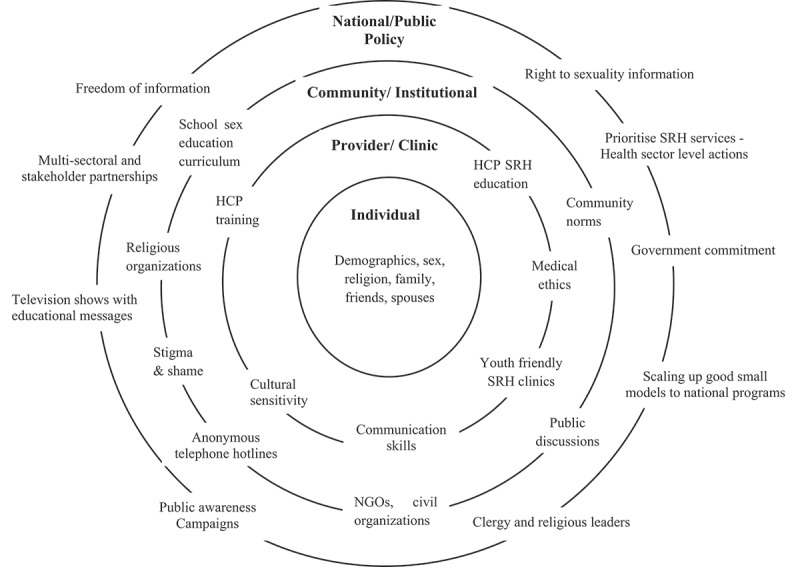
Socioecological model appraising the factors influencing men’s sexual and reproductive health with focus on cultural-specific considerations.

HCP: health-care professional; SRH: sexual and reproductive health; NGO: Non-governmental Organization

Minimizing the cultural restraints around men’s SRH is a formidable challenge, particularly in conservative societies. Thus, culture-specific effort considerations will inevitably need multi-layered approaches encompassing common values and mutual concerns, culture, gender norms, barriers, religious values and sensitivities [64]. Such efforts should be implemented at several levels: national/public policy; community/institutional; provider/clinic; the individual concerned, as well as his family, friends, and partners [64]. Based on the socioecological model framework [30], collectively Figure 3 and Table 3 depict the possible way forward pertaining to these levels, as well level-specific opportunities for improvement.

**Table 3.** Men’s SRH in MENA: culture-specific solutions.

Domain/Level	Possible way forward: Opportunities
National/public policy	Country-wide, multi-sectoral partnerships and policies required to respond to calls for more government commitment to translate small scale models in MENA to national programs to improve young peoples’ reproductive health welfare [129]. MENA nations should prioritise SRH as pressing issue [109]. Television shows, mass media channels and Internet reach large audience, incorporating education points could increase sexual health communication/awareness [89,130]
Community/institutional	To emphasize that sexuality is a healthy topic that can be discussed, sexuality education needs to engage widely with those who are consulted on sexual issues e.g. teachers, marriage counselors, and religious scholars [131]. Educational campaigns may use different outlets e.g. schools, medical training, and social media to reach targeted audience [55,132]. Our review revealed that school-based peer education interventions were effective in improving HIV transmission and prevention knowledge, decreasing misconceptions and stigma and discrimination towards PLWHA. Education among school adolescents possible in conservative settings, if interventions are addressed in a culturally sensitive manner, and key stakeholders are involved [55]
	At the wider community level, findings from our review could be used to inform the development of a culturally sensitive sexual health education for Muslim youth. As many Muslims depend on guidance from religious leaders, capitalizing NGOs and civil organizations to involve such leaders while promoting sexual health education, as well as community involvement ensures that materials are clearly understood and accepted in conservative Muslim cultures
Provider/clinic	Overcoming cultural constraints requires facilitating dialogue between patients and physicians on sensitive issues such as ED as this helps patients seek proper and safe medical advice [12]. In addition, specialized and confidential adolescent/youth friendly clinic services could prove useful [130,133–135]. Country-specific examples currently exist that could be implemented across MENA. For instance, Egypt and Oman have confidential SRH and HIV anonymous telephone hotlines that disproportionately serve young people [136,137]. Likewise, summer caravans toured Morocco providing condoms/voluntary confidential HIV testing/counselling to young people [138]; and Tunisia has adolescent health clinics open to unmarried young people [139].
	Particularly for HCP, the present review emphasized the importance of reminding and increasing HCP’s awareness about medical ethics and rules that regulate their profession, regardless of HCP’s judgments, and to uphold healthcare human rights [33,35]. In addition, HCP training and education needs to incorporate sexuality issues, with specialized sexuality lectures/workshops related to responsibility, cultural ethical issues, confidence, and positive practice of sexual counselling, using culturally relevant role-playing/small group discussions to provide HCP the opportunities to analyze their beliefs toward sexual counselling, and discourse sensitive issues using effective communication skills/appropriate language, hence asserting positive sexual counselling practice in real life [74,112,140]
Individual	Efforts required to increase men’s cultural acceptance to SRH issues e.g. to remind clients that birth control is not prohibited by Islamic law; emphasize that men have vital role in preventing unintended pregnancies [141]; highlight that effective contraception use in Muslim world is important for religio-cultural reasons forbidding termination of pregnancies [142–144]; and that men might need to share equally the responsibility of fertility regulation [145]. Cooperation and compliance of patient as well as patient’s spouses, family, and friends are vital for successful change to overcome cultural barriers. This requires increased awareness of the diverse cultural attitudes, beliefs, and values about health issues as this could affect care-seeking

PLWHA: people living with HIV/AIDS; HCP: health care professionals; ED: erectile dysfunction; HIV: Human Immunodeficiency Virus; SRH: Sexual & reproductive health; MENA: Middle East and North Africa.

## Conclusion

Socio-cultural aspects are deeply rooted across MENA population groups including HCPs, students, general public, and patients with negative impact on the perceptions and dealings pertaining to men’s SRH issues including HIV, reproduction and sexuality. Five cultural notions were observed, namely gender equality; religious prohibitions and misinterpretations; sexual rights and taboos; masculinity ideals and manhood; and large families and consanguinity. In parallel, pockets of cultural changes are emerging in MENA, positively influencing men’s SRH domains. Future efforts would require concerted actions across national/public policymakers, community/institutional stakeholders, healthcare providers, and individuals to address cultural taboos and sensitivities, as well as religious misconceptions. Such efforts might require multipronged strategies that could embrace health, education, and national policies. The observed paucity of socio-cultural research on men’s SRH across MENA generally, and some nations in particular, calls for efforts for more high-quality evidence spanning more countries.

## Supplementary Material

Supplemental Material

## Data Availability

Data are available upon request.

## Data availability statement

Data is available upon reasonable request.

## References

[1] UNFPA (United Nations Fund for Population Activities) Arab States. Sexual and reproductive health. 2022 [cited 2023 Jul 21]. Available from: https://arabstates.unfpa.org/en/topics/sexualandreproductivehealth

[2] The World Bank. Population, male (% of total population) - Middle East & North Africa. (WA): The World Bank; 2022 [cited 2023 Jul 20]. Available from: https://data.worldbank.org/indicator/SP.POP.TOTL.MA.ZS?locations=ZQ

[3] OECD iLibrary. Youth at the centre of government action: a review of the Middle East and North Africa. 2023 [cited 2023 Jul 20]. Available from: https://www.oecd-ilibrary.org/sites/3ced02bf-en/index.html?itemId=/content/component/3ced02bf-en#:~:text=Across%20most%20of%20the%20Middle,OECD%20countries%20(Figure%201.1)

[4] Farrag NS, Cheskin LJ, Farag MK. A systematic review of childhood obesity in the Middle East and North Africa (MENA) region: prevalence and risk factors meta-analysis. Adv Pediatr Res. 2017;4:8. doi: 10.12715/apr.2017.4.8 29354689 PMC5773115

[5] Alkhasawneh E, McFarland W, Mandel J, et al. Insight into Jordanian thinking about HIV: knowledge of Jordanian men and women about HIV prevention. J Assoc Nurses AIDS Care. 2014;25(1):e1–9. doi: 10.1016/j.jana.2013.06.001 Epub 2013 Oct 14. PMID: 24135312. 24135312

[6] World Health Organization. Defining sexual health: report of a technical consultation on sexual health. 2006 [cited 2023 May 10]. Available from: http://www.who.int/reproductivehealth/topics/gender_rights/defining_sexual_health.pdf

[7] El-Sakka AI. Erectile dysfunction in Arab countries. Part I: prevalence and correlates. Arab J Urol. 2012;10(2):97–103. doi: 10.1016/j.aju.2012.01.004 26558010 PMC4442907

[8] Mumtaz GR, Riedner G, Abu-Raddad LJ. The emerging face of the HIV epidemic in the Middle East and North Africa. Curr Opin HIV AIDS. 2014;9(2):183–191. doi: 10.1097/COH.0000000000000038 24445372 PMC3921267

[9] UNAIDS. Global HIV and AIDS statistics. The joint united nations programme on HIV/AIDS. 2018 [cited 2022 Mar 20]. Available from: http://aidsinfounaidsorg/

[10] Tabazah S. 103 HIV/AIDS cases registered in Jordan in 2016. The Jordan time. 2017 [cited 2023 Jul 17]. Available from: http://wwwjordantimescom/news/local/103-hivaids-cases-registered-jordan-2016

[11] Filemban SM, Yasein YA, Abdalla MH, et al. Prevalence and behavioral risk factors for STIs/hiv among attendees of the ministry of health hospitals in Saudi Arabia. J Infect Dev Ctries. 2015;9(4):402–408. doi: 10.3855/jidc.5964 25881530

[12] Abdulah Al Turki Y. Should an inquiry about sexual health, as a reflection of vascular health, be part of routine physicals for young men? Results from an outpatient study. Int J Impot Res. 2009;21(6):362–365. doi: 10.1038/ijir.2009.36 19693020

[13] Sexual relations between spouses: as Islamic perspective - let us correct our Islamic faith. 2015 [cited 2023 Aug 10]. Available from: http://www.correctislamicfaith.com/sexualpracticeinislam.htm

[14] Akhu-Zaheya LM, Masadeh AB. Sexual information needs of Arab-Muslim patients with cardiac problems. Eur J Cardiovasc Nurs. 2015;14(6):478–485. doi: 10.1177/1474515115597353 26201826

[15] Adib SM, Akoum S, El-Assaad S, et al. Heterosexual awareness and practices among Lebanese male conscripts. East Mediterr Health J. 2002;8(6):765–775. doi: 10.26719/2002.8.6.765 PMID: 15568454. 15568454

[16] The World Bank. Characterizing the HIV/AIDS epidemic in the Middle East and North Africa. Time for strategic action. (WA): The World Bank; 2010.

[17] El Ansari W, Arafa M, Elbardisi H, et al. Scoping review of sexual and reproductive healthcare for men in the MENA (Middle East and North Africa) region: a handful of paradoxes? BMC Publ Health. 2023;23(1):564. doi: 10.1186/s12889-022-14716-2 PMC10040932 36973770

[18] Shand T, Marcell AV. Engaging men in sexual and reproductive health. Oxford Res Encycl Of Global Public Health. 2021. doi: 10.1093/acrefore/9780190632366.013.215

[19] Page MJ, McKenzie JE, Bossuyt PM, et al. The PRISMA 2020 statement: an updated guideline for reporting systematic reviews. BMJ. 2021;372:n71. doi: 10.1136/bmj.n71 33782057 PMC8005924

[20] Inocian EP, Nolfi DA, Felicilda-Reynaldo RFD, et al. Bariatric surgery in the Middle East and North Africa: narrative review with focus on culture-specific considerations. Surg Obes Relat Dis. 2021;17(11):1933–1941. doi: 10.1016/j.soard.2021.06.015 34332910

[21] Leininger M. Transcultural care principles, human rights, and ethical considerations. J Transcultural Nurs. 1991;3(1):21–23. doi: 10.1177/104365969100300105 1747208

[22] United Nations Population Fund. Sexual & reproductive health. 2022 [cited 2023 Jun 20]. Available from: https://www.unfpa.org/sexual-reproductive-health

[23] Canadian Paediatric Society. A guide for health professionals working with immigrant and refugee children and youth. 2023 [cited 2023 Aug 2]. Available from: https://kidsnewtocanada.ca/culture/How-Culture-Influences-Health

[24] Weiss G. A scientific concept of culture. Am Anthropol. 1973;75(5):1376–1413. doi: 10.1525/aa.1973.75.5.02a00130

[25] Pew Research Center. The future of world religions: population growth projections, 2010­-2050. 2015 [cited 2023 Jun 24]. Available from: http://www.pewforum.org/2015/04/02/religious-projections-2010-2050

[26] Stonawski M, Skirbekk V, Hackett C. Global population projections by religion: 2010–2050. In: Yearbook of international religious demography. Brill; 2015. p. 99–116.

[27] Hackett C, Stonawski M, Potančoková M. The future size of religiously affiliated and unaffiliated populations. DemRes. 2015;32:829–842. doi: 10.4054/DemRes.2015.32.27

[28] De Souza M, Rymarz R. The perceptions of some Australian Coptic students of the influences on their religious development. J Beliefs Values. 2003;24(1):67–74. doi: 10.1080/1361767032000053006

[29] Germov J. Theorising health: major theoretical perspectives in health sociology. In: Germov J, editor. Second opinion: an introduction to health sociology. 3rd ed. Melbourne: Oxford University Press; 2005. p. 28–50.

[30] McLeroy KR, Bibeau D, Steckler A, et al. An ecological perspective on health promotion programs. Health Educ Q. 1988;15(4):351–377. doi: 10.1177/109019818801500401 PMID: 3068205. 3068205

[31] Memish ZA, Filemban SM, Bamgboyel A, et al. Knowledge and attitudes of doctors toward people living with HIV/AIDS in Saudi Arabia. J Acquir Immune Defic Syndr. 2015;69(1):61–67. doi: 10.1097/QAI.0000000000000550 25642972

[32] Kabbash IA, Abo Ali EA, Elgendy MM, et al. Hiv/aids-related stigma and discrimination among health care workers at tanta university hospitals, Egypt. Environ Sci Pollut Res Int. 2018;25(31):30755–30762. doi: 10.1007/s11356-016-7848-x 27752955

[33] Abdelrahman I, Lohiniva AL, Kandeel A, et al. Learning about barriers to care for people living with HIV in Egypt: a qualitative exploratory study. J Int Assoc Provid AIDS Care. 2015 Mar-Apr;14(2):141–147. doi: 10.1177/2325957413488180 23792709

[34] Bashir F, Ba Wazir M, Schumann B, et al. The realities of HIV prevention. A closer look at facilitators and challenges faced by HIV prevention programmes in Sudan and Yemen. Glob Health Action. 2019;12(1):1659098. doi: 10.1080/16549716.2019.1659098 31496422 PMC6746302

[35] Lohiniva AL, Benkirane M, Numair T, et al. HIV stigma intervention in a low-hiv prevalence setting: a pilot study in an Egyptian healthcare facility. AIDS Care. 2016;28(5):644–652. doi: 10.1080/09540121.2015.1124974 26717980

[36] Al-Iryani B, Raja’a YA, Kok G, et al. HIV knowledge and stigmatization among adolescents in Yemeni schools. Int Q Community Health Educ. 2009;30(4):311–320. doi: 10.2190/IQ.30.4.c 21273165

[37] Al-Mazrou YY, Abouzeid MS, Al-Jeffri MH. Knowledge and attitudes of paramedical students in Saudi Arabia toward HIV/AIDS. Saudi Med J. 2005;26(8):1183–1189. 16127510

[38] Al-Rabeei NA, Dallak AM, Al-Awadi FG. Knowledge, attitude and beliefs towards HIV/AIDS among students of health institutes in Sana’a city. East Mediterr Health J. 2012;18(3):221–226. doi: 10.26719/2012.18.3.221 22574474

[39] Badahdah AM. Stigmatization of persons with HIV/AIDS in Saudi Arabia. J Transcult Nurs. 2010;21(4):386–392. doi: 10.1177/1043659609360873 20592063

[40] Gańczak M, Barss P, Alfaresi F, et al. Break the silence: HIV/AIDS knowledge, attitudes, and educational needs among Arab university students in United Arab Emirates. J Adolesc Health. 2007;40(6):572.e1–8. doi: 10.1016/j.jadohealth.2007.01.011 17531765

[41] Haroun D, El Saleh O, Wood L, et al. Assessing knowledge of, and attitudes to, HIV/AIDS among university students in the United Arab Emirates. PLOS ONE. 2016;11(2):e0149920. doi: 10.1371/journal.pone.0149920 26913902 PMC4767799

[42] Al Tall YR, Mukattash TL, Sheikha H, et al. An assessment of HIV patient’s adherence to treatment and need for pharmaceutical care in Jordan. Int J Clin Pract. 2020;74(7):e13509. doi: 10.1111/ijcp.13509 32279382

[43] Kulane A, Owuor JOA, Sematimba D, et al. Access to HIV care and resilience in a long-term conflict setting: a qualitative assessment of the experiences of living with diagnosed HIV in Mogadishu, Somali. Int J Environ Res Public Health. 2017;14(7):721. doi: 10.3390/ijerph14070721 28678166 PMC5551159

[44] Al-Serouri AW, Takioldin M, Oshish H, et al. Knowledge, attitudes and beliefs about HIV/AIDS in Sana’a, Yemen. East Mediterr Health J. 2002;8(6):706–715. doi: 10.26719/2002.8.6.706 PMID: 15568447. 15568447

[45] Al-Serouri AW, Anaam M, Al-Iryani B, et al. AIDS awareness and attitudes among Yemeni young people living in high-risk areas. East Mediterr Health J. 2010;16(3):242–250. doi: 10.26719/2010.16.3.242 PMID: 20795436. 20795436

[46] Arheiam A, El Tantawi M, Al-Ansari A, et al. Arab dentists’ refusal to treat HIV positive patients: a survey of recently graduated dentists from three Arab dental schools. Acta Odontol Scand. 2017;75(5):355–360. doi: 10.1080/00016357.2017.1316867 Epub 2017 Apr 21. Erratum in: Acta Odontol Scand 2017; 75(8): 634. 28431481

[47] Mahfouz AA, Alakija W, Al-Khozayem Aa, et al. Knowledge and attitudes towards AIDS among primary health care physicians in the asir region, Saudi Arabia. J R Soc Health. 1995;115(1):23–25. doi: 10.1177/146642409511500108 7738977

[48] Fido A, Al Kazemi R. Survey of HIV/AIDS knowledge and attitudes of Kuwaiti family physicians. Fam Pract. 2002 Dec;19(6):682–684. doi: 10.1093/fampra/19.6.682 12429674

[49] Hassan ZM, Wahsheh MA. Knowledge and attitudes of Jordanian nurses towards patients with HIV/AIDS: findings from a nationwide survey. Issues Ment Health Nurs. 2011;32(12):774–784. doi: 10.3109/01612840.2011.610562 22077750

[50] Shah S, Elgalib A, Al-Wahaibi A, et al. Knowledge, attitudes and practices related to HIV stigma and discrimination among healthcare workers in Oman. Sultan Qaboos Univ Med J. 2020;20(1):e29–e36. doi: 10.18295/squmj.2020.20.01.005 32190367 PMC7065692

[51] Aunon FM, Wagner GJ, Maher R, et al. An exploratory study of HIV risk behaviors and testing among male sex workers in Beirut, Lebanon. Soc Work Publ Health. 2015;30(4):373–384. doi: 10.1080/19371918.2014.979274 PMC4562212 25950906

[52] AlMuzaini AA, Yahya AS, Ellepola AN, et al. HIV/AIDS: dental assistants’ self-reported knowledge and attitudes in Kuwait. Int Dent J. 2015;65(2):96–102. doi: 10.1111/idj.12136 25345503 PMC9376531

[53] Lohiniva AL, Kamal W, Benkirane M, et al. HIV stigma toward people living with HIV and health providers associated with their care: qualitative interviews with community members in Egypt. J Assoc Nurses AIDS Care. 2016;27(2):188–198. doi: 10.1016/j.jana.2015.11.007 26718817

[54] Al-Jabri AA, Al-Abri JH. Knowledge and attitudes of undergraduate medical and non-medical students in Sultan Qaboos University toward acquired immune deficiency syndrome. Saudi Med J. 2003;24(3):273–277. PMID: 12704503. 12704503

[55] Al-Iryani B, Basaleem H, Al-Sakkaf K, et al. Evaluation of a school-based HIV prevention intervention among Yemeni adolescents. BMC Publ Health. 2011;11(1):279. doi: 10.1186/1471-2458-11-279 PMC3112119 21548968

[56] Al-Owaish R, Moussa MA, Anwar S, et al. Knowledge, attitudes, beliefs, and practices about HIV/AIDS in Kuwait. AIDS Educ Prev. 1999;11(2):163–173. PMID: 10214499. 10214499

[57] Ghazeeri G, Zebian D, Nassar AH, et al. Knowledge, attitudes and awareness regarding fertility preservation among oncologists and clinical practitioners in Lebanon. Hum Fertil (Camb). 2016 Jun;19(2):127–133. doi: 10.1080/14647273.2016.1193636 27376977

[58] Arafa MA, Rabah DM. Attitudes and practices of oncologists toward fertility preservation. J Pediatr Hematol Oncol. 2011;33(3):203–207. doi: 10.1097/MPH.0b013e3182068047 PMID: 21336166. 21336166

[59] Rabah DM, Wahdan IH, Merdawy A, et al. Oncologists’ knowledge and practice towards sperm cryopreservation in Arabic communities. J Cancer Surviv. 2010;4(3):279–283. doi: 10.1007/s11764-010-0140-z Epub 2010 Jul 23. PMID: 20652434. 20652434

[60] Melaibari M, Shilbayeh S, Kabli A. University students’ knowledge, attitudes, and practices towards the national premarital screening program of Saudi Arabia. J Egypt Public Health Assoc. 2017;92(1):36–43. doi: 10.21608/EPX.2018.6648 29924926

[61] Jaffer YA, Afifi M, Al Ajmi F, et al. Knowledge, attitudes and practices of secondary-school pupils in Oman: II. Reproductive health. East Mediterr Health J. 2006;12(1–2):50–60 PMID: 17037221. 17037221

[62] Eshra DK, Dorgham LS, el-Sherbini AF. Knowledge and attitudes towards premarital counselling and examination. J Egypt Public Health Assoc. 1989;64(1–2):1–15. PMID: 2520141. 2520141

[63] Khalaf I, Abu Moghli F, Froelicher ES. Youth-friendly reproductive health services in Jordan from the perspective of the youth: a descriptive qualitative study. Scand J Caring Sci. 2010;24(2):321–331. doi: 10.1111/j.1471-6712.2009.00723.x 20233355

[64] Karim SI, Irfan F, Saad H, et al. Men’s knowledge, attitude, and barriers towards emergency contraception: a facility based cross-sectional study at King Saud University Medical City. PLoS One. 2021;16(4):e0249292. doi: 10.1371/journal.pone.0249292 33901184 PMC8075244

[65] Ghazal-Aswad S, Zaib-Un-Nisa S, Rizk DE, et al. A study on the knowledge and practice of contraception among men in the United Arab Emirates. J Fam Plann Reprod Health Care. 2002;28(4):196–200. doi: 10.1783/147118902101196559 12419060

[66] Mustafa MA, Mumford SD. Male attitudes towards family planning in Khartoum, Sudan. J Biosoc Sci. 1984;16(4):437–449. doi: 10.1017/s0021932000015273 6490682

[67] Ismael AS, Sabir Zangana JM. Knowledge, attitudes and practice of condom use among males aged (15-49) years in Erbil governorate. Glob J Health Sci. 2012;4(4):27–36. doi: 10.5539/gjhs.v4n4p27 22980338 PMC4776938

[68] Hamdan-Mansour AM, Malkawi AO, Sato T, et al. Men’s perceptions of and participation in family planning in Aqaba and Ma’an governorates, Jordan. East Mediterr Health J. 2016;22(2):124–132. doi: 10.26719/2016.22.2.124 27180740

[69] Spindler E, Bitar N, Solo J, et al. Jordan’s 2002 to 2012 fertility stall and parallel USAID investments in family planning: lessons from an assessment to guide future programming. Glob Health Sci Pract. 2017;5(4):617–629. doi: 10.9745/GHSP-D-17-00191 29284697 PMC5752608

[70] Barakat M, Thiab S, Thiab S, et al. Knowledge and perception regarding the development and acceptability of male contraceptives among pharmacists: a mixed sequential method. Am J Mens Health. 2022;16(1):15579883221074855. doi: 10.1177/15579883221074855 35135388 PMC8832602

[71] Sait M, Aljarbou A, Almannie R, et al. Knowledge, attitudes, and perception patterns of contraception methods: cross-sectional study among Saudi males. Urol Ann. 2021;13(3):243–253. doi: 10.4103/UA.UA_42_20 Epub 2021 Jul 14. 34421259 PMC8343273

[72] Farrag S, Hayter M. A qualitative study of Egyptian school nurses’ attitudes and experiences toward sex and relationship education. J Sch Nurs. 2014;30(1):49–56. doi: 10.1177/1059840513506941 24106180

[73] Bdair IA, Maribbay GL. Perceived knowledge, practices, attitudes and beliefs of Jordanian nurses toward sexual health assessment of patients with coronary artery diseases. Sex Disabil. 2020;38(3):491–502. doi: 10.1007/s11195-020-09639-y

[74] Abu Ali RM, Abed MA, Khalil AA, et al. A survey on sexual counseling for patients with cardiac disease among nurses in Jordan. J Cardiovasc Nurs. 2018;33(5):467–473. doi: 10.1097/JCN.0000000000000472 PMID: 29601371. 29601371

[75] Abdulmohsen MF, Abdulrahman IS, Al-Khadra AH, et al. Physicians’ knowledge, attitude and practice towards erectile dysfunction in Saudi Arabia. East Mediterr Health J. 2004;10(4–5):648–654. doi: 10.26719/2004.10.4-5.648 16335658

[76] Kapoor NR, Langer A, Othman A, et al. Healthcare practitioners experiences in delivering sexual and reproductive health services to unmarried adolescent clients in Jordan: results from a cross-sectional survey. BMC Health Serv Res. 2022;22(1):31. doi: 10.1186/s12913-021-07415-y 34986832 PMC8734334

[77] Salameh P, Zeenny R, Salamé J, et al. Attitudes towards and practice of sexuality among university students in Lebanon. J Biosoc Sci. 2016;48(2):233–248. doi: 10.1017/S0021932015000139 26040203

[78] Naal H, Abboud S, Harfoush O, et al. Examining the attitudes and behaviors of health-care providers toward LGBT patients in Lebanon. J Homosex. 2020;67(13):1902–1919. doi: 10.1080/00918369.2019.1616431 31125288

[79] Elmahy AG. Reaching Egyptian gays using social media: a comprehensive health study and a framework for future research. J Homosex. 2018;65(13):1867–1876. doi: 10.1080/00918369.2017.1395658 29053437

[80] U.S. Department of Health and Human Services. National institutes of health, national cancer institute. Theory at a glance: a guide for health promotion practice. 2nd ed. Bethesda (MD): National Institutes of Health; 2005.

[81] World Health Organization. Sexual health and its linkages to reproductive health: an operational approach. Geneva, Switzerland: WHO; 2017 [cited 2023 Apr 2]. Available from: http://apps.who.int/iris/bitstream/handle/10665/258738/9789241512886-eng.pdf;jsessionid=A20D089FFCBCA9F97264F98E57881DA1?sequence=1

[82] Gausman J, Othman A, Al-Qotob R, et al. Health care professionals’ attitudes towards youth-friendly sexual and reproductive health services in Jordan: a cross-sectional study of physicians, midwives and nurses. Reprod Health. 2021;18(1):84. doi: 10.1186/s12978-021-01137-4 33882951 PMC8059015

[83] Al-Shdayfat NM, Green G. Reflections on sex research among young Bedouin in Jordan: risks and limitations. Cult Health Sex. 2012;14(1):101–111. doi: 10.1080/13691058.2011.626871 22085369

[84] Torres López TM, Reynaldos Quinteros C, Lozano González AF, et al. Concepciones culturales del VIH/Sida de adolescentes de Bolivia, Chile y México. Rev Saude Publica. 2010;44(5):820–829. doi: 10.1590/S0034-89102010000500007 20877920

[85] EuroMed Info. How culture influences health beliefs. 2021 [cited 2023 May 26]. Available from: https://www.euromedinfo.eu/how-culture-influences-health-beliefs.html/

[86] Mateus MD, Santos JQ, Mari JJ. Popular conceptions of schizophrenia in Cape Verde, Africa. Rev Bras Psiquiatr. 2005;27(2):101–107. doi: 10.1590/S1516-44462005000200006 15962133

[87] Rosén I. The impact of culture on health – a study of risk perception on unhealthy lifestyles in Babati town, Tanzania [Bachelor’s thesis]. Södertörn University, School of Natural Sciences, Technology and Environmental Studies; 2015 [cited 2023 May 18]. Available from: https://www.diva-portal.org/smash/get/diva2:824535/FULLTEXT01.pdf

[88] Gamaoun R. Knowledge, awareness and acceptability of anti-hpv vaccine in the Arab states of the Middle East and North Africa region: a systematic review. East Mediterr Health J. 2018;24(6):538–548. doi: 10.26719/2018.24.6.538 30079949

[89] Alomair N, Alageel S, Davies N, et al. Factors influencing sexual and reproductive health of Muslim women: a systematic review. Reprod Health. 2020;17(1):33. doi: 10.1186/s12978-020-0888-1 32138744 PMC7059374

[90] Abu Habib L, Abdel Khalik Z. Sexual and reproductive health in the Arab region. Arab states civil society organizations and feminists network. 2021 [cited 2023 May 5]. Available from: https://arabstates.unwomen.org/sites/default/files/Field%20Office%20Arab%20States/Attachments/2021/07/SRHR-Policy%20Paper-EN.pdf

[91] Bassey SA, Bubu NG. Gender inequality in Africa: a re-examination of cultural values. Cogito - Multidiscip Res J. 2019;3:21–36.

[92] Promundo. Men in the Middle East and North Africa (MENA) at a crossroads, reveals ground-breaking, multi-country study on the state of gender equality in the region. 2017 [cited 2023 Jun 21]. Available from: https://promundoglobal.org/2017/05/02/men-in-the-middle-east-and-northafrica-mena-at-a-crossroads-reveals-ground-breaking-multi-country-study-on-the-stateof-gender-equality-in-the-region/

[93] Statistica. Percentage of population in the Middle East and North Africa that belonged to major religious groups in 2010, by religion. 2014 [cited 2023 Jul 27]. Available from: https://www.statista.com/statistics/374759/population-in-middle-east-north-africa-by-religion/

[94] World Atlas. Christianity in the Middle East: countries with the highest Christian population. 2017 [cited 2023 Mar 5]. Available from: https://www.worldatlas.com/articles/christianity-in-the-middle-east-countries-with-the-highest-christian-population.html

[95] Runkel G. Sexual morality of christianity. J Sex Marital Ther. 1998;24(2):103–122. doi: 10.1080/00926239808404924 9611690

[96] Adamczyk A, Pitt C. Shaping attitudes about homosexuality: the role of religion and cultural context. Soc Sci Res. 2009;38(2):338–351. doi: 10.1016/j.ssresearch.2009.01.002 19827178

[97] Joint United Nations Programme on HIV/AIDS and World Health Organization UNAIDS/WHO. Report on the global AIDS epidemic. Geneva, Switzerland: WHO; 2008. p. 3–10.

[98] Krishna V, Bhatti R, Chandra P, et al. Unheard voices: experiences of families living with HIV/AIDS in India. Contemp Fam Ther. 2005;27(4):483–506. doi: 10.1007/s10591-005-8235-9

[99] Reyes-Estrada M, Varas-Díaz N, Martínez-Sarson MT. Religion and HIV/AIDS stigma: considerations for the nursing profession. New Sch Psychol Bull. 2015;12(1):48–55. doi: 10.2307/3583350 PMC4744372 26858806

[100] Zou J, Yamanaka Y, John M, et al. Religion and HIV in Tanzania: influence of religious beliefs on HIV stigma, disclosure, and treatment attitudes. BMC Publ Health. 2009;9(1):975. doi: 10.1186/1471-2458-9-75 PMC2656538 19261186

[101] Parker W. The impact of religious belief and stigma on people living with HIV/AIDS: a study in Cravenby, Cape Town. University of the Western Cape; 2014 [cited 2023 May 3]. Available from: https://etd.uwc.ac.za/xmlui/bitstream/handle/11394/4332/parker_w_ma_arts_2014.pdf?sequence=1&isAllowed=y

[102] Meldrum RM, Liamputtong P, Wollersheim D. Sexual health knowledge and needs: young Muslim women in Melbourne, Australia. Int J Health Serv. 2016;46(1):124–140. doi: 10.1177/0020731415615313 26536914

[103] Amraei S, Abedi P, Nikbakht R, et al. Does infertility stress impair sexual function in infertile women and men? A cross-sectional study in Iran. Front Med (Lausanne). 2022;9:896538. doi: 10.3389/fmed.2022.896538 35814743 PMC9258717

[104] Francesca E. AIDS in contemporary Islamic ethical literature. Med Law. 2002;21(2):381–394. PMID: 12184613. 12184613

[105] Hammoud MM, White CB, Fetters MD. Opening cultural doors: providing culturally sensitive healthcare to Arab American and American Muslim patients. Am J Obstet Gynecol. 2005;193(4):1307–1311. doi: 10.1016/j.ajog.2005.06.065 16202719

[106] Oshi DC, Nakalema S, Oshi LL. Cultural and social aspects of HIV/AIDS sex education in secondary schools in Nigeria. J Biosoc Sci. 2005;37(2):175–183. doi: 10.1017/s0021932004006820 15768772

[107] Visser MJ. Life skills training as HIV/AIDS preventive strategy in secondary schools: evaluation of a large-scale implementation process. Sahara J. 2005;2(1):203–216. doi: 10.1080/17290376.2005.9724843 17601024

[108] Halstead JM. Muslims and sex education. J Moral Educ. 1997;26(3):317–330. doi: 10.1080/0305724970260306

[109] Roudi-Fahimi F, El-Feki S. Facts of life: youth sexuality and reproductive health in the Middle East and North Africa. Popul Reference Bureau. 2011.

[110] Ashraah MM, Gmaian I, Al-Shudaifat S. Sex education as viewed by Islam education. EJSR. 2013;95(1):5Y16.

[111] Ozdemir L, Akdemir N. Nurses’ knowledge and practice involving patients’ resuming sexual activity following myocardial infarction: implications for training. Aust J Adv Nurs. 2008;26(1):42–52. doi: 10.37464/2008.261.1791

[112] Jaarsma T, Strömberg A, Fridlund B, et al. UNITE research group. Sexual counselling of cardiac patients: nurses’ perception of practice, responsibility and confidence. Eur J Cardiovasc Nurs. 2010;9(1):24–29. doi: 10.1016/j.ejcnurse.2009.11.003 Epub 2009 Dec 11. PMID: 20005178. 20005178

[113] Goossens E, Norekvål TM, Faerch J, et al. Sexual counselling of cardiac patients in Europe: culture matters. Int J Clin Pract. 2011;65(10):1092–1099. doi: 10.1111/j.1742-1241.2011.02756.x PMID: 21923848. 21923848

[114] Doherty S, Byrne M, Murphy AW, et al. Cardiac rehabilitation staff views about discussing sexual issues with coronary heart disease patients: a national survey in Ireland. Eur J Cardiovasc Nurs. 2011;10(2):101–107. doi: 10.1016/j.ejcnurse.2010.05.002 Epub 2010 Jun 1. PMID: 20684891. 20684891

[115] Akdolun N, Terakye G. Sexual problems before and after myocardial infarction: patients’ needs for information. Rehabil Nurs. 2001;26(4):152–158. doi: 10.1002/j.2048-7940.2001.tb01939.x 12035583

[116] Salonia A, Capogrosso P, Clementi MC, et al. Is erectile dysfunction a reliable indicator of general health status in men? Arab J Urol. 2013;11(3):203–211. doi: 10.1016/j.aju.2013.07.008 26558083 PMC4443011

[117] Maulana AO, Krumeich A, Van Den Borne B. Emerging discourse: Islamic teaching in HIV prevention in Kenya. Cult Health Sex. 2009;11(5):559–569. doi: 10.1080/13691050902792771 19437176

[118] Mhirsi Z. Epidemiology of sexually transmitted infections in Arab countries. 2nd congress of the federation of Arab societies of clinical microbiology and infectious diseases, 24-26 May 2012. 2012 [cited 2023 Jun 17]. Available from: https://www.infectiologie.org.tn/pdf_ppt_docs/congres/165313122722.pdf

[119] Serour GI. Attitudes & cultural perspective on infertility and its alleviation in the middle East area. In: Vayena E, Rowep P, and Griffin D, editors. Current practices and controversies in assisted reproduction. Geneva, Switzerland: Report of a WHO (World Health Organization) meeting; 2002. p. 41–49.

[120] Albakr A, Arafa M, Elbardisi H, et al. Premature ejaculation: an investigative study into assumptions, facts and perceptions of patients from the middle East (PEAP STUDY). Arab J Urol. 2021;19(3):303–309. doi: 10.1080/2090598X.2021.1948159 34552781 PMC8451653

[121] Abouelenin M. Gender, resources, and intimate partner violence against women in Egypt before and after the Arab Spring. Violence Against Women. 2022;28(2):347–374. doi: 10.1177/1077801221992877 33656937 PMC8721615

[122] Alghabashi MT, Guthrie B. Systematic review of human immunodeficiency virus (HIV) knowledge measurement instruments used on the Arabian Peninsula. BMC Res Notes. 2015;8(1):646. doi: 10.1186/s13104-015-1614-x 26537121 PMC4634190

[123] Albanghali MA, Othman BA. A cross-sectional study on the knowledge of sexually transmitted diseases among young adults living in Albaha, Saudi Arabia. Int J Environ Res Public Health. 2020;17(6):1872. doi: 10.3390/ijerph17061872 32183110 PMC7142563

[124] El Hasbani G, Jawad ASM, Uthman I. Rheumatology research output in the Arab world: despite the challenges. Reumatismo. 2022;74(3). doi: 10.4081/reumatismo.2022.1520 36580063

[125] El Ansari W, Afifi Soweid RA, Jabbour S. Geography of biomedical publications. Lancet. 2004;363(9407):489–490. doi: 10.1016/S0140-6736(04)15498-6 14962532

[126] Youthpolicy.org. Middle East and North Africa: youth facts. 2016 [cited 2023 Apr 10]. Available from: https://www.youthpolicy.org/mappings/regionalyouthscenes/mena/facts/

[127] Population Reference Bureau. Population trends and challenges in the Middle East and North Africa. 2001 [cited 2023 Feb 11]. Available from: https://www.prb.org/resources/population-trends-and-challenges-in-the-middle-east-and-north-africa/

[128] Chahil-Graf R, Madani N. Women, culture and the HIV epidemic in MENA. J Int AIDS Soc. 2014;17(1):19074. doi: 10.7448/IAS.17.1.19074 24629846 PMC3955763

[129] DeJong J, El-Khoury G. Reproductive health of Arab young people. BMJ. 2006;333(7573):849–851. doi: 10.1136/bmj.38993.460197.68 17053245 PMC1618449

[130] Seetharaman S, Yen S, Ammerman SD. Improving adolescent knowledge of emergency contraception: challenges and solutions. Open Access J Contracept. 2016;7:161–173. doi: 10.2147/OAJC.S97075 29386948 PMC5683156

[131] Moreira ED, Brock G, Glasser DB, et al. Help-seeking behaviour for sexual problems: the global study of sexual attitudes and behaviors. Int J Clin Pract. 2005;59(1):6–16. doi: 10.1111/j.1742-1241.2005.00382.x 15707457

[132] Alwafi HA, Meer AMT, Shabkah A, et al. Knowledge and attitudes toward HIV/AIDS among the general population of Jeddah, Saudi Arabia. J Infect Public Health. 2018;11(1):80–84. doi: 10.1016/j.jiph.2017.04.005 28579268

[133] Tylee A, Haller DM, Graham T, et al. Youth-friendly primary-care services: how are we doing and what more needs to be done? The Lancet. 2007;369(9572):1565–1573. doi: 10.1016/S0140-6736(07)60371-7 17482988

[134] Pathfinder International. Making reproductive health services youth friendly. (WA): Focus on Young Adults; 1999.

[135] World Health Organization. Quality assessment guidebook: a guide to assessing health services for adolescent clients. Geneva: WHO; 2009.

[136] Egyptian Ministry of Health and Population. Report on AIDS hot-line 1 January - 31 December 2001. Cairo: MHP; 2002.

[137] Oman National AIDS Programme. HIV/AIDS hotline records. Oman: Ministry of Health; 2006.

[138] UNAIDS. Summer caravan drives forward HIV prevention efforts in Morocco. Press briefing. 2006 Sep 4 [cited 2023 May 4]. Available from: www.unaids.org/en/MediaCentre/PressMaterials/FeatureStory/20060901-morocco.asp

[139] World Health Organization. Growing in confidence: programming for adolescent health and development. Lessons from eight countries. Geneva: WHO; 2002.

[140] Röndahl G, Innala S, Carlsson M. Nursing staff and nursing students’ attitudes towards hiv-infected and homosexual hiv-infected patients in Sweden and the wish to refrain from nursing. J Adv Nurs. 2003;41(5):454–461. doi: 10.1046/j.1365-2648.2003.02553.x 12603570

[141] Marcell AV, Waks AB, Rutkow L, et al. What do we know about males and emergency contraception? A synthesis of the literature. Perspect Sex Reprod Health. 2012;44(3):184–193. doi: 10.1363/4418412 22958663

[142] Shapiro GK. Abortion law in Muslim-majority countries: an overview of the Islamic discourse with policy implications. Health Policy Plan. 2014;29(4):483–494. doi: 10.1093/heapol/czt040 23749735

[143] El Hamri N. Approaches to family planning in Muslim communities. J Fam Plann Reprod Health Care. 2010;36(1):27–31. doi: 10.1783/147118910790291019 20067669

[144] Al-Matary A, Ali J. Controversies and considerations regarding the termination of pregnancy for foetal anomalies in Islam. BMC Med Ethics. 2014;15(1):10. doi: 10.1186/1472-6939-15-10 24499356 PMC3943453

[145] Ringheim K. Factors that determine prevalence of use of contraceptive methods for men. Stud Fam Plann. 1993;24(2):87–99. doi: 10.2307/2939202 PMID: 8511809. 8511809

