# Supplementary material for: Breaking the silence – systematic review of the socio-cultural underpinnings of men’s sexual and reproductive health in Middle East and North Africa (MENA): A handful of taboos?
Source: Arab J Urol. 2024 Aug 13;23(1):16–32. doi: 10.1080/20905998.2024.2387511 (PMC11703040; doi:10.1080/20905998.2024.2387511)
Supplement: Supplemental Material [file TAJU_A_2387511_SM4964.docx]

**Supplementary Box 1: Search terms used in the current systematic review**

| Male, Men, Male, Sexual Health, Reproductive Health, Sexual and Reproductive Health, SRH, Sexual and Reproductive Health Rights, SRHR, Sexual Rights, Consanguinity, Sexual Function, Sexual Dysfunction, Erectile Dysfunction, Sexual Satisfaction, Men's Health, Sex Offenses, Sexual Violence, Gender-Based Violence, Family Planning, Contraceptive, Contraception, Condom, Infertility, Fertility, Genital Diseases, Sexually Transmitted Diseases, STDs, Sexually Transmitted Infections, STIs, Chlamydia, Gonorrhoea, Gonorrhea, Syphilis, Trichomonas, Trichomoniasis, Herpes Genitalis, Human Papilloma Virus, Genital Warts, HIV, Human Immunodeficiency Virus, AIDS, Acquired Immunodeficiency Syndrome, PLWHA, People Living with HIV and AIDS, Homosexuality, MSM, Men Having Sex With Men, Transgender, Transsexual, Homosexual, LGBT  Health Services Accessibility, Health Facilities, Health Services, Delivery Of Health Care, Preventive Health Services, Clinic, Hospital, Primary Care, Health Care Providers, Students, Adolescents, Young Adults, Public, General Population, Patients, Culture, Cultural, Social, Socio-Cultural, Sociocultural, psychosocial, Taboo, Myth, Norm, Social Norm, Cultural Values, Beliefs, philosophy, kinship, Lifeways, Politics, Economy, Education, Religion, Religious, Islam, Muslim, Christianity, Christian, Jewish, Jews, Shame, Stigma, Avoidance, Avoid, Masculinity, Attitude, Satisfaction, Satisfied, Acceptance, Accept, Perception, Perceive, Expectation, Expect, Experience, Quality, Trust  MENA, Middle East and North Africa, Middle East, Arabic Countries, Arabian, Algeria, Algerian, Djibouti, Djiboutian, Egypt, Egyptian, Iraq, Iraqi, Jordan, Jordanian, Kuwait, Kuwaiti, Lebanon, Lebanese, Libya, Libyan, Mauritania, Mauritanian, Morocco, Moroccan, Palestine, Palestinian, Saudi Arabia, Saudi Arabian, KSA, Sudan, Sudanese, Syria, Syrian, Tunis, Tunisian, United Arab Emirates, UAE, U.A.E., Yemen, Yemeni |
| --- |
